# Whole-Genome Sequencing of Two Extensively Drug-Resistant Mycobacterium tuberculosis Isolates from India

**DOI:** 10.1128/MRA.00007-19

**Published:** 2019-02-14

**Authors:** Syed Beenish Rufai, Sarman Singh

**Affiliations:** a Division of Clinical Microbiology and Molecular Medicine, All India Institute of Medical Sciences, New Delhi, India; b Clinical Microbiology and Molecular Medicine, All India Institute of Medical Sciences, Bhopal, India; Georgia Institute of Technology

## Abstract

The emergence of extensively drug-resistant tuberculosis (XDR-TB) presents a considerable challenge and a public health concern due to the high mortality rate of this disease. Whole-genome sequencing (WGS) of XDR-TB isolates is thus essential for understanding the mechanism of drug resistance. Here we report whole-genome sequences of two XDR-TB strains of two lineages from India.

## Abstract (precis)

The emergence of extensively drug-resistant tuberculosis (XDR-TB) presents a considerable challenge and a public health concern due to the high mortality rate of this disease. Whole-genome sequencing (WGS) of XDR-TB isolates is thus essential for understanding the mechanism of drug resistance.

## ANNOUNCEMENT

Next-generation sequencing (NGS) of whole genomes has paved the way for understanding the mechanisms of drug resistance at the genomic level (1). This information can lead to timely and appropriate treatment strategies to reduce further transmission of the resistant strains. Two indigenous strains of Mycobacterium tuberculosis (both from sputum samples from pulmonary TB patients) were isolated from the North-Western Provinces of India and given the laboratory identification (ID) numbers L-182 and L-31, respectively. Both sputum samples were processed using the *N*-acetyl-l-cysteine-sodium citrate-NaOH (NALC-NaOH) method. For each isolate, 500 µl of the decontaminated sample was inoculated in Bactec-MGIT960 per the manufacturer's instructions (Becton Dickinson, Sparks, MD, USA) (2). Identification and characterization of the M. tuberculosis isolates was done by using multiplex PCR as published earlier (3). The first- and second-line drug susceptibility testing of the above M. tuberculosis cultures, which identified the two isolates as XDR-MTB, was performed by using a Bactec MGIT-960 system (BD, Franklin Lakes, NJ, USA) according to the manufacturer’s instructions (4). The DNA of these isolates was extracted by using the chloroform-isoamyl-alcohol method (5, 6) followed by molecular characterization by application of a spoligotyping technique using a commercial kit (Ocimum Biosolutions, India). The spoligotyping method, which was done per the manufacturer’s instructions (7), identified the genetic lineages of the M. tuberculosis isolates. The spoligotyping analysis confirmed that one isolate belonged to the Beijing (L-182) strain lineage and the other one (L-31) to the Central Asian strain lineage.

Whole-genome sequencing of both isolates was performed using the Ion Torrent PGM platform on an Ion 316v2 chip. Briefly, the genomic DNA library was prepared using an Ion Xpress Plus fragment library kit and an Ion Xpress barcode adapter kit. Size selection of a library for targeted read length was carried out by using a size fractionation method with 2% E‐Gel SizeSelect gel according to the manufacturer’s instructions (Ion Torrent, Life Technologies). A barcoded target library of 200 bp was prepared for L-182 and L-31, which resulted in totals of 856,225 and 854,481 reads with mean read lengths of 181 bp and 167 bp, respectively. Template preparation from the diluted library was performed using an Ion PGM template OT2 200 kit (Ion Torrent, Life Technologies). Template amplification and enrichment were performed using the Ion OneTouch 2 system, which consists of two Ion OneTouch 2 instruments and the Ion OneTouch ES enrichment system (Ion Torrent). The whole-genome sequencing was performed using an Ion PGM machine (Ion Torrent). The Torrent Suite software plugin v 5.2.1 (an integral part of the Ion Torrent PGM platform) filtered out low-quality reads using a base caller and also trimmed away barcode sequences, extra bases, and short sequences (–trim-qual-cutoff, 16) (Ion Torrent). An unaligned BAM file was passed to a Torrent Mapping Alignment Program (TMAP) for alignment of data (8).

*De novo* assembly was then performed using an ion plugin assembler, the St. Petersburg genome assembler (SPAdes) (v 3.1.0), with k-mers of defined length (default settings). The overall numbers of contigs for L-182 and L-31 were 326 and 283, of which 210 and 166 were ≥500 bp, respectively. The total lengths of contigs of ≥500 bp were 4,293,547 and 4,272,039 bp, and the *N*_50_ contig lengths were 45,790 and 56,210 bp for L-182 and L-31, respectively. The annotation of genomes was performed using Rapid Annotation using Subsystem Technology (RAST), which provided total sizes of 42,88,394 bp and 43,11,779 bp, with 65.4 and 65.3% GC contents and coding sequences of 4,737 and 4,813 for L-182 and L-31 isolates, respectively (9).

### Data availability.

This genome project has been deposited in GenBank under the accession numbers NDYV00000000 for L-182 and NDYU00000000 for L-31. Raw sequencing data sets have been registered in the NCBI SRA database under the accession numbers SRR8426999 for L-182 and SRR8427345 for L-31. The versions outlined in the present paper are the first versions for both of the accession numbers.
